# A structured overview of simultaneous component based data integration

**DOI:** 10.1186/1471-2105-10-246

**Published:** 2009-08-11

**Authors:** Katrijn Van Deun, Age K Smilde, Mariët J van der Werf, Henk AL Kiers, Iven Van Mechelen

**Affiliations:** 1 SymBioSys, Katholieke Universiteit Leuven, Leuven, Belgium; 2 Biosystems data analysis, Swammerdam Institute for Life Sciences, University of Amsterdam, Amsterdam, The Netherlands; 3 TNO, Quality of Life, Zeist, The Netherlands; 4 Heymans Institute, University of Groningen, Groningen, The Netherlands

## Abstract

**Background:**

Data integration is currently one of the main challenges in the biomedical sciences. Often different pieces of information are gathered on the same set of entities (e.g., tissues, culture samples, biomolecules) with the different pieces stemming, for example, from different measurement techniques. This implies that more and more data appear that consist of two or more data arrays that have a shared mode. An integrative analysis of such coupled data should be based on a simultaneous analysis of all data arrays. In this respect, the family of simultaneous component methods (e.g., SUM-PCA, unrestricted PCovR, MFA, STATIS, and SCA-P) is a natural choice. Yet, different simultaneous component methods may lead to quite different results.

**Results:**

We offer a structured overview of simultaneous component methods that frames them in a principal components setting such that both the common core of the methods and the specific elements with regard to which they differ are highlighted. An overview of principles is given that may guide the data analyst in choosing an appropriate simultaneous component method. Several theoretical and practical issues are illustrated with an empirical example on metabolomics data for *Escherichia coli *as obtained with different analytical chemical measurement methods.

**Conclusion:**

Of the aspects in which the simultaneous component methods differ, pre-processing and weighting are consequential. Especially, the type of weighting of the different matrices is essential for simultaneous component analysis. These types are shown to be linked to different specifications of the idea of a fair integration of the different coupled arrays.

## Background

Recently, technological developments have led to a situation where data analysts in different domains face data that are more and more complex. A special case of complex data are coupled data that consist of different data matrices for the same set of variables or experimental units. In systems biology, an example of matrices sharing the same set of variables is the study of the expression profile of a certain organism (e.g., *Saccharomyces cerevisae*) on the basis of on the one hand different microarray compendia that can be downloaded from public repositories, and on the basis of, on the other hand, ChIP-chip or motif data [1,2]. An example of data matrices with shared experimental units are metabolomics data (e.g., the metabolome of *Escherichia coli*) gathered from different fermentations using mass spectrometry (MS) with different MS data sets being available from different separation methods (e.g., gas chromatography and liquid chromatography [3]). In the first example each of the data matrices provides information on the same transcriptome and in the second example on the same set of metabolites, with some parts of the information being common for the different data matrices and some parts being specific: For example, gas chromatography mass spectrometry (GC/MS) and liquid chromatography MS (LC/MS) in general measure both a few classes of common compounds and many classes of compounds that are measured by one of the two methods only [3,4].

A major challenge for researchers dealing with such coupled data, is to represent them in such a way that both shared and specific information as contained in the different data matrices is captured (with all information in question pertaining to variance within each of the matrices under study). For example, in the case of coupled gene expression and ChIP-chip data one may wish to retrieve modules of genes that have the same transcription factors and that are co-regulated under the same conditions, which is common information as contained in the transcriptome and ChIP-chip data matrices; in the metabolomics example, a coupled data analysis of gas and liquid chromatography MS data should allow to highlight the classes of compounds that are measured by both separation methods, as well as those that are measured by only one of them.

Several tools are available that can be used for the analysis of coupled data. Here we will focus on methods that simultaneously extract components from all data blocks. Examples of such methods include SUM-PCA [5], unrestricted PCovR (Gurden: Multiway covariates regression, unpublished), SCA-P [6], multiple factor analysis [7], and STATIS [8]. Whereas all these methods are based on the idea of a simultaneous component extraction they have been developed independently in different disciplines (including chemometrics and psychometrics) and rely on different terminologies and mathematical frameworks. As a consequence, comparing them is not straightforward. The primary objective of this paper is to provide a structured overview in which all the methods fit, and to highlight their common core and particularities.

The paper starts by introducing some terminology to delineate the types of data to which the methods are applicable; then, a general framework is introduced that encompasses all the different simultaneous component methods and that frames them mathematically into a principal components setting. Then, each of the methods is discussed with respect to this framework. An application is presented on simultaneous components analyses of gas and liquid chromatography MS data; in this application we compare the results obtained by applying the different methods and discuss how to interpret the results obtained by one of the methods (multiple factor analysis).

## Methods

### Some terminology

**Figure 1:**
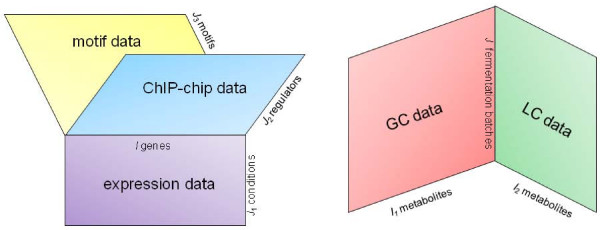
**Illustrations of coupled data**. Illustrations of coupled two-way two-mode data that share a single mode: In the left panel three data matrices (gene expression data, motif data, and ChIP-chip data) share the row mode (genes); in the right panel two data blocks (gas chromatography and liquid chromatography mass spectrometry data) share the column mode (fermentation batches).

In this paper, we are interested in multiblock (or multiset) data consisting of at least two two-way two-mode data blocks that have one mode in common. Two-way two-mode data denote rectangular data matrices where the term mode is used to indicate one of the sets of units that underlie the data, namely the set of row elements or the set of column elements. For example, a condition and a gene mode may underlie gene expression data while a condition and a metabolite mode may underlie mass spectrometry data. Often the mode containing the experimental units (e.g., conditions), is called the object mode while the mode containing the variables (e.g., genes, metabolites) is called the variable mode. Usually the data are organized such that the rows of the data matrix correspond to the different objects and the columns to the different variables. Here, we will consider collections of coupled data matrices for which the shared, or linked, mode is the same for all couples of data matrices. Figure 1 gives a graphical representation of two cases of such data: In the left panel, three data matrices that share the row mode are represented and in the right panel two data matrices that are linked by the column mode. It might seem a trivial issue to distinguish between these two cases as by transposing the data matrices one may move from one case to the other, but in the next section it will become clear that the results obtained from a method for data with a coupled object (row) mode may be different from those obtained with a method for coupled variable (column) data.

### A structural overview of Simultaneous component methods

In this section, first a general framework is presented that encompasses all simultaneous component methods; then each of the methods is discussed with respect to the general framework.

#### The general framework

The aspects that constitute the general framework are 1) the data to which the methods apply, 2) pre-processing steps included in the methods, 3) a general mathematical model for the data, 4) the objective function that is needed to estimate the model parameters, 5) identification constraints to obtain a unique solution for the parameters, and 6) the algorithmic strategy used to derive the model parameters (on the basis of the objective function, subject to the identification constraints).

We will follow the notation introduced in [9], denoting vectors and matrices by bold upper and lower case letters respectively and using indexes for which the cardinality is indicated by the capital of the letter used to run the index. For example, the different data matrices will be indexed by *k*, with *k *running from 1 to *K*.

##### The data

As discussed in section 2, simultaneous component methods are applicable to data consisting of at least two two-way two-mode data matrices that have one mode in common. The common mode can be either the object or the variable mode.

##### Pre-processing

The data might be pre-processed in order to correct for irrelevant differences between variables or matrices. For example, when different measurement units are used to measure the variables the resulting differences in offset and scale might be accounted for by centering each variable (such that the resulting variable has mean zero) and by scaling to sum of squares or to range one [3] (the combined operation of centering and scaling to sum of squares one is usually named autoscaling or standardizing); see also [10]. Coupled matrices may also differ in size such that the results of a particular statistical analysis may be dominated by the largest matrices; to correct for this effect, a possible strategy is to scale each data matrix to sum of squares one.

##### The model

Assume *K *data matrices **X**_*k *_containing the scores of *I*_*k *_objects on *J*_*k *_variables. Modeling each of these by a principal components structure with *R *components gives,

(1)

with *w*_*k *_(*w*_*k *_≥ 0) denoting a prespecified block weight, **T**_*k *_an *I*_*k *_× *R *matrix of component scores, **P**_*k *_the *J*_*k *_× *R *matrix of loadings, and **E**_*k *_the *I*_*k *_× *J*_*k *_matrix of residuals. In the general model, the fact that the *K *matrices are coupled is taken explicitly into account by subjecting (1) to the constraint that the model matrix that relates to the common mode is the same for all data matrices. In case of a common row mode (with *I*_1 _= ... = *I*_*K *_= *I*) this introduces the constraint **T**_1 _= ... = **T**_*K *_= **T **such that the matrices are modeled by

(2)

with **T **denoting the common component scores and **P**_*k *_the matrix-specific loadings. Similarly, for a common column mode (*J*_1 _= ... = *J*_*K *_= *J*), the constraint is imposed that **P**_1 _= ... = **P**_*K *_= **P**, so that the model becomes

(3)

with **P**_1 _= ... = **P**_*K *_= **P **the common loadings. This gives the general model

(4)

Except for the equality constraint on the common component scores in (2) or on the loadings in (3), identification constraints (e.g., orthogonality or orthonormality) might be applicable but the latter do not affect the scores reproduced by the model, **T**_*k*_.

##### Objective function

To estimate the model parameters **T**_*k *_and **P**_*k*_, the following optimization criterion is introduced:

(5)

under the restriction that either **T**_1 _= ... = **T**_*K *_= **T **(common object mode) or **P**_1 _= ... = **P**_*K *_= **P **(common variable mode).

##### Identification constraints

In general it holds for any nonsingular matrix **B **that if **T**_*k *_and **P**_*k *_are solutions of (5), **T**_*k*_**B **and **P**_*k*_**B**^-1 ^are solutions too (e.g., **B **can be a rotation matrix). Examples of identification constraints often encountered in practice are that the component scores and loadings are expressed with respect to orthogonal axes in the direction of the highest variance (principal axes orientation) and that the component scores or loadings are orthonormal.

##### Model estimation

Optimal parameters that minimize (5) can be obtained either by a singular value decomposition (SVD) of the concatenated weighted data matrices or by a two-step approach in which first the common structure is found by an eigendecomposition of the sum of cross-product matrices and second the *K *companion matrices are found by means of a suitable regression analysis. Both strategies can be used for data with a coupled object (row) mode as well as for data with a coupled variable (column) mode and they result in the same solution. We refer to the additional file (see additional file 1: Estimation.pdf) for details on both estimation procedures and a proof of their equivalence.

###### Specific simultaneous component methods

In the previous section we have set up a general framework for a simultaneous component representation of data that consist of at least two matrices sharing a common mode. Here, we will show how five published methods are specializations of the general framework. These are SUM-PCA [5], unrestricted principal covariates regression (unrestricted PCovR see [11] and also Gurden: Multiway covariates regression, unpublished), multiple factor analysis (MFA [7,12]), STATIS [8], and SCA-P [6]).

The general framework includes six aspects: (1) the data, (2) pre-processing, (3) the model, (4) the objective function, (5) the identification constraints, and (6) the algorithmic strategy. We will discuss the published methods with respect to the specific choices they make for the first, second, third, and fifth aspect. The fourth aspect (the objective function) is not discussed because it is the same for all methods; the sixth aspect (the algorithmic strategy) is not discussed either, because the choice made with respect to this aspect does not affect the obtained solution.

#### Specific simultaneous component methods

In the previous section we have set up a general framework for a simultaneous component representation of data that consist of at least two matrices sharing a common mode. Here, we will show how five published methods are specializations of the general framework. These are SUM-PCA [5], unrestricted principal covariates regression (unrestricted PCovR see [11] and also Gurden: Multiway covariates regression, unpublished), multiple factor analysis (MFA [7,12]), STATIS [8], and SCA-P [6]).

The general framework includes six aspects: (1) the data, (2) pre-processing, (3) the model, (4) the objective function, (5) the identification constraints, and (6) the algorithmic strategy. We will discuss the published methods with respect to the specific choices they make for the first, second, third, and fifth aspect. The fourth aspect (the objective function) is not discussed because it is the same for all methods; the sixth aspect (the algorithmic strategy) is not discussed either, because the choice made with respect to this aspect does not affect the obtained solution.

##### SUM-PCA

A first published method that fits within our general framework is SUM-PCA. A confusing element though, is that the name SUMPCA has been used for two different methods: this paragraph bears on SUM-PCA (with a hyphen) proposed in the chemometric literature [5] and not on SUMPCA proposed in the psychometric literature [13,14]; we will come back to the latter method when discussing SCA-P.

• Data

SUM-PCA [5] was developed for data that are linked in the row mode.

• Pre-processing

The pre-processing steps consist of first autoscaling the data per variable and secondly, scaling each data block to sum of squares equal to one [5].

• Model

SUM-PCA models the *K *two-way data blocks by the following mathematical structure,

(6)

which implies pre-specified weights *w*_*k *_= 1. Note, however, that the pre-processing step of a scaling of each block to sum of squares one applied to autoscaled data, is equivalent to using weights on the autoscaled data; this means blocks with more variables are downweighted more than blocks with fewer variables in order to avoid that larger blocks dominate the solution.

• Identification constraints

The SUM-PCA model (6) is estimated under the identification constraints of a principal axes orientation and orthonormality of the common component scores: **T**^*T*^**T **= **I**. The latter constraint is unusual in chemometrics where it is common practice to have orthonormal loadings. However, it implies that the method finds exactly the same global scores as CPCA-W, a method that extracts the components in a sequential way (for a proof of the equivalence, see [5]).

##### Unrestricted PCovR

• Data

Principal covariates regression (PCovR) was proposed for the analysis of data consisting of a matrix of dependent variables **X**_1 _and a matrix of independent variables **X**_2 _for the same set of objects [11].

• Pre-processing

In [11], different options are mentioned for pre-processing on the level of the variables, including centering, scaling and autoscaling.

• Model

Principal covariates regression represents the data by means of the following model,

(7)

with the common component scores **T**_1 _being restricted to belong to the column space of **X**_1 _and with 0 ≤ *β *≤ 1. Such a restriction does not fit within the general framework as outlined above; yet, in the more general context of multiway covariates regression, also an unrestricted model has been proposed in which this restriction was dropped (Gurden: Multiway covariates regression, unpublished); clearly, the unrestricted PCovR model fits within the general framework. The pre-specified weight is determined by a strategy that minimizes the cross-validation error for the prediction of **X**_2_; this introduces some asymmetry in the treatment of **X**_1 _and **X**_2_. Both simulation studies (Gurden: Multiway covariates regression, unpublished) and empirical results [15] yielded unsatisfying results for the proposed cross-validation approach because it results in *β*-values close to zero or one, implying that all weight is placed on a single matrix.

• Identification constraints

In [11], it is suggested to constrain the common component scores to be column-wise orthonormal: **T**^*T*^**T **= **I**.

##### MFA

• Data

Multiple Factor Analysis or MFA [7,12,16], also known as Analyse Factorielle Multiple or AFM, was proposed in the French literature as a method for the analysis of data consisting of several sets of variables for the same group of subjects (common object mode). Recently, the method was applied to integrate distinct omics data [17].

• Pre-processing

The variables are supposed to be autoscaled.

• Model

Multiple Factor Analysis is based on the following model,

(8)

with *σ*_*k*1 _being the largest singular value of **X**_*k*_. The choice of the inverted *σ*_*k*1_'s as the matrix-specific weights is motivated by the fact that in this way two corrections take place at once: one for differences in the number of variables and one for the redundancy of the information contained by the data matrices. This can be understood by observing the following properties of the eigenvalues: (1) the size of the matrix can be measured by  (which equals *J*_*k *_for autoscaled variables); (2) redundancy can be measured by the proportion of VAF by the first component, . So, matrix **X**_*k *_can be corrected for size and redundancy by

(9)

which is the correction used by MFA. Note that scaling the matrices (e.g., to unit sum of squares) is of no influence on the MFA results as the singular value of *f *times **X**_*k *_equals *fσ*_*k *_and the first step of MFA is to divide each block by this singular value.

• Identification constraints

MFA estimates the parameters under a principal axes constraint and orthonormality of the component scores: **T**^*T*^**T **= **I**.

##### STATIS

• Data

STATIS was proposed in the French literature [8] (see also [13] for an English description of the method). In both publications, three-way data are used but the authors note that the method is also applicable to coupled two-way data matrices with a common object mode.

• Pre-processing

In [8], nothing is mentioned on pre-processing. Centering and scaling to unit variance are mentioned as options of the STATIS (ACT) method in [18]; a weighting to account for differences in size of the matrices is described in [19].

• Model

STATIS is based on the following model,

(10)

with *a*_*k *_being the weight associated to the *k*th data matrix. These weights are obtained from the first component of the PCA of a matrix which is derived from the data in the following way: 1) Derive the cross-product matrices **S**_*k *_=  (note that for vectors of autoscaled object scores, this would be the matrix of correlations between objects). 2) Construct the matrix **F **of size *N*^2 ^× *K *by inserting the vectorized matrix **S**_*k*_, formally written as vec(**S**_*k*_), in the *k*th column. The weights *a*_*k *_are the loadings on the first principal component of **F **and can be obtained from the first right singular vector of **F **or from an eigendecomposition of the matrix **S **with values  (note that the element on the intersection of row *k *and column *k' *of the matrix **F**^*T*^**F **equals vec (**S**_*k*_)^*T *^vec(**S**_*k'*_) so **S **= **F**^*T*^**F**). Larger weights can be expected for: (a) data matrices with larger values (the values on the diagonal of the cross product matrix **S**_*k *_will be larger for such matrices and thus the loading associated to the kth column of **F**, vec(**S**_*k*_)), (b) larger matrices, (c) data matrices with more covariation between the vectors of object scores, and (d) matrices with more similar cross product matrices to other matrices (*K > *2). The latter property is the motivation for the proposed weighting strategy: STATIS wants to find a *compromise *of the different cross-product matrices **S**_*k*_. This idea of a compromise or a consensus is prominent in the analysis of three-way data and underlies Generalized Procrustes analysis (GPA) and Generalized Canonical Correlation analysis [20]. See [21,22] for a comparison of STATIS and GPA.

• Identification constraints

STATIS looks for simultaneous components with a principal axes orientation and under the restriction of orthonormal loadings (**P**_*conc *_= **I**, with **P**_*conc *_representing the matrix of concatenated block specific loadings).

##### SCA-P

• Data

In the psychometric literature, SCA-P was proposed as a method for the analysis of multiple data matrices obtained by measuring the same set of variables in different groups; the variable mode is therefore considered to be the common mode [6]. The acronym SCA-P stands for simultaneous component analysis with a common pattern matrix, where "pattern matrix" is a psychometric term for "loading matrix".

• Pre-processing

In the original applications of SCA-P, where the main interest is to account for variation within the matrices **X**_*k*_, it is recommended to autoscale each variable per data block [6]. Another proposed strategy is to center each variable per matrix and to scale the variables to sum of squares ∑_*k*_*I*_*k *_for the concatenated data, this is over matrices [23]. The latter strategy preserves the variability within the matrices **X**_*k*_. In the remainder of the paper we will refer to SCA-P with a variable-wise autoscaling per block.

• Model

The following model is used to structure the data,

(11)

with **P **a common loading matrix. The different blocks are not explicitly weighted but larger blocks will not contribute more to the common components given that the variable mode is common and that each variable has been autoscaled: This can be understood by observing that **P **can be found by the eigendecomposition of  with **R**_*k *_the matrix of correlations between the variables in data matrix *k*. Indeed, this is the method denoted as SUMPCA [13,14], hence SCA-P is equivalent to SUMPCA and also to Levin's method [24] that minimizes

(12)

• Identification constraints

SCA-P looks for simultaneous components that have a prinicipal axes orientation and orthonormal component scores, **T**_*conc*_= **I**, with **T**_*conc *_representing the matrix of concatenated block specific loadings).

### Reflection on the general framework

We presented a general framework for simultaneous component methods and showed how several published methods fit within the framework. In this section we will reflect on the comparability of the simultaneous component results when making a specific choice for different aspects of the general framework. In Table 1 a summary overview of the published methods discussed in the methods section is given in function of the specific choices they make with respect to the four aspects as discussed above: 1) the mode that is considered common (object or variable), 2) the pre-processing steps, 3) the pre-specified matrix-specific weights, and 4) the identification constraints. To arrive at a deeper understanding of the relations between the different published methods below we will first consider each of these aspects and the different choices made by the published methods with regard to them; second, we will discuss more in detail those aspects for which the choices are consequential for the obtained results.

**Table 1:** Characterization of published simultaneous component methods in function of the general framework.

	Common mode	Pre-processing	Matrix-specific weights	Identification constraint
SUM-PCA	object	Variables: auto-scaling Matrices: scaled to sum of squares one	All *w*_*k *_= 1	Principal axes **T**^*T*^**T **= **I**
unr. PCovR	object	Variables: auto-scaling	Minimize cross- validation error	Principal axes **T**^*T*^**T **= **I**
MFA	object	Variables: auto-scaling	Inverse of largest singular value	Principal axes **T**^*T*^**T **= **I**
STATIS	object		Compromise weights	Principal axes **P**_*conc *_= **I**
SCA-P	variable	Variables: auto-scaling	All *w*_*k *_= 1	Principal axes **T**_*conc *_= **I**

#### Structured overview of the methods

A first aspect concerns the labeling of the common mode as the object or as the variable mode. Ignoring for a moment what happens on the level of pre-processing and weighting but taking into account whether the orthonormality restriction is imposed on the common mode or on the other mode, changing the label of the common mode from object to variable or vice versa makes that STATIS turns into SCA-P or vice versa. Second, with respect to pre-processing, all methods except STATIS autoscale each variable. Furthermore, SUM-PCA additionally also scales each matrix to unit sum of squares (alternatively this can also be considered a matrix-specific weighting strategy). Third, except for SCA-P, all methods use a distinct matrix-specific weighting strategy (note that as indicated above SUM-PCA can be considered to do this weighting via the pre-processing step). Fourth, all methods use a principal axis identification constraint but differ in their choice of putting an orthonormality constraint either on the common structure (SUM-PCA, unr. PCovR, and MFA) or on the concatenated structure (STATIS and SCA-P); as discussed previously, however, this affects the scaling of the components only, and yields the same reconstructed data.

#### Consequential differences between the methods

Next, one may wonder for which of the aspects as discussed above the choices may be consequential for the obtained results. A first such aspect is the type of pre-processing, with all methods except STATIS relying on a variable-wise autoscaling. This means that the solutions obtained with STATIS can be dominated by the variables with the largest sums of squares; for that reason, these solutions can be very different from the results as obtained with the other methods. A second possibly consequential aspect is the matrix-specific weighting strategy. This can strongly influence the obtained results, with as an extreme all weight being put on a particular matrix which results in a structure of the common mode (**T **or **P**) that equals the structure obtained from the separate component analysis of that matrix. Note that in case the *R *dominant directions of the different data matrices span approximately the same subspace, a matrix-specific weighting will have little impact on the obtained results (Escoufier's RV coefficient can be used to measure this dependency between the data matrices [25,26]). Third, although the labeling of the common mode has no direct consequential effect, it may have important indirect consequences through what happens on the level of pre-processing. Indeed, methods that perform autoscaling do this in the direction of the variables: Labeling the common mode as 'variables' results in autoscaling in the direction of the common mode while labeling it as 'objects' results in autoscaling in the other direction.

Furthermore, matrix-specific weighting operates differently for autoscaled data with a common object mode than for autoscaled data with a common variable mode. To understand the latter, let the row elements (which may pertain to either objects or variables) be common, such that the common structure (**T **or **P**) can be derived from  when the row mode is the variable mode **X**_*k*_ is a correlation matrix and when the row mode is the object mode it will be a cross-product matrix. Now, as the size of a correlation does not depend on the sample size, in case of a common variable mode larger matrices will not necessarily have a larger contribution; on the other hand entries of cross-product matrices may take larger values in case of larger matrices. As a consequence, in case of a common object mode, larger matrices may contribute more to the sum  hence in such a case, unlike in a case with a common variable mode, a downweighting of larger matrices may be desired. Note that in the description of the methods used here, we are strict in the choices made on the level of pre-processing. The methods, however, could also be defined on the basis of the matrix-specific weighting strategy apart from any pre-processing strategy. Then, the labelling of the common mode is not consequential (such that SCA-P can also be used for data that are coupled in the variable mode while SUM-PCA, unr. PCovR, MFA, and STATIS can also be used for data coupled in the object mode).

#### Reflection on pre-processing and weighting

The proposed pre-processing and weighting strategies reflect data-analytic concerns to correct for possible disturbing factors on the level of the variables and on the level of the matrices. First, on the level of the variables a disturbing factor may stem from different variables being measured by different measurement techniques or with different measurement scales. Often such differences may be irrelevant from a substantive perspective but they are essential and may distort the results, which may necessitate a suitable type of preprocessing. Now STATIS as original published [8], unlike all other published methods does not autoscale variables; therefore, in case of irrelevant differences between variables in measurement scale, STATIS may not be used or only in combination with a preliminary centering, scaling, or autoscaling of the data as proposed in later publications on the method [18,19].

Second, on the level of the different matrices a major concern may lead to combine them in a fair way. Different possible principles of fairness can be considered; an overview is given in Table 2. A first (naive) principle may be to give equal weight to all matrices (which may be realized through a mere concatenation of the data). More sophisticated principles imply the use of matrix-specific weights to correct for possible unwanted dominance of some matrices. A first such principle is to attach more weight to smaller matrices in order to avoid that large matrices dominate the solution. Second, more weight may be given to matrices that contain more heterogeneous information in order to avoid that the solution is dominated by redundancies. Third, one may wish to give more weight to matrices containing more stable predictive information, in order to avoid that the solution is steered by particularities that cannot be replicated. Fourth, one may wish to give more weight to matrices that have more in common with other matrices to avoid that idiosyncracies dominate the solution. The different published methods aim at specific principles (see Table 2).

**Table 2:** Principles to realize a fair integration of different data matrices.

Principle	Methods aiming at this principle
Same weight for all matrices (naive approach)	SCA-P
More weight for smaller matrices	SUM-PCA, MFA
More weight for less redundant matrices	MFA
More weight for matrices with more stable predictive information	PCovR
More weight for matrices that share more information with other matrices (*K *> 2)	STATIS

It can be useful to apply these principles of fairness in a flexible way. For example, different principles may be combined when there are different reasons for unwanted dominance of a matrix. This is the case for the weighting strategy underlying MFA; another example is a modified weighting strategy for STATIS that corrects for differences in sizes between the matrices and also gives more weight to matrices that have more in common with other matrices [21,22]. Also, different principles can be used for different data matrices. For example, suppose that a particular matrix is very noisy, that all matrices have equal sum of squares, and that interest is in avoiding redundancies. In the noisy (i.e., extremely heterogenous) block all singular values will be almost equal such that applying the principle of more weight for the less redundant matrices will give most weight to the noisy data block. In such a case one may consider to use the principle of more weight for the matrices with more stable predictive information in order to give less weight to this particular block, and to additionally use the principle of avoiding redundancies on the other blocks.

## Results

In this Section we will apply each of the existing methods to an example biological dataset. First the data are described, then each of the methods is applied and the solution obtained with MFA is interpreted.

### Description of the data

The phenylalanine production in the Escherichia coli NST 74 and a wild type strain was studied with the aim to identify bottlenecks in the production of this compound [3]. Metabolomes were screened at different fermentation times and obtained under various environmental conditions using both gas chromatography (GC) and liquid chromatography (LC) in combination with mass spectrometry (MS). In general GC/MS and LC/MS methods are known to detect different classes of chemical compounds, although some classes are detected by both methods [4]. The data consist of two coupled data matrices: a GC/MS matrix with the measurements of 144 metabolites (including 13 intermediates of the phenylalanine biosynthesis route) and a LC/MS matrix with the measurements of 44 metabolites, both for the same 28 samples of E. coli (common object mode); no metabolite was measured on both platforms, hence, there is no trivial overlap. In the data considered here, only those metabolites that were detected in at least 20 percent of the experiments were used; furthermore, the data were manually curated and normalized as described by [27]. Measurement values below the detection threshold were set equal to one half of the smallest detected value [3]: 687 (17 percent) of the GC measurements and 34 (3 percent) of the LC measurements were below the limit of detection. Due to a lot of values below the detection limit for a few metabolites in the GC data, some extreme outliers were observed. To deal with skewness and asymmetry, all values were log-transformed. No influential outliers were found among the log-transformed values.

### Application of different simultaneous component methods

The different published simultaneous component methods were applied to the coupled GC/MS – LC/MS data. Due to the asymmetry associated to the cross-validation procedure in PCovR, we report two unrestricted PCovR analyses denoted as PCovR GC and PCovR LC with the former denoting a leave-one-out crossvalidation based on using the observed scores in the LC data to reproduce the scores of the GC data and the latter a crossvalidation that uses the observed GC data to reproduce the LC data. We also report the analysis based on the unweighted but variable-wise autoscaled data. This can be considered to be SCA-P applied to the transposed versions of the data matrices.

An overview of the relative matrix-specific weights, calculated as the matrix-specific sum of squares divided by the sum of squares of the concatenated data, obtained with the different simultaneous component methods is given in Table 3: For ease of comparison, these are expressed as the sum of squares of the specific weighted matrix divided by the total sum of squares over all weighted matrices. For the first method reported (PCovR GC) all weight is put on the GC matrix; such extreme weights could be expected on the basis of what has been reported in the literature (Gurden: Multiway covariates regression, unpublished and [15]). Also STATIS uses extreme weights with almost all weight put on the GC matrix; this can be mainly attributed to the fact that the GC matrix is much larger than the LC matrix. The relative weights obtained for SCA-P purely reflect the size difference, with the GC matrix being approximately three times as large as the LC matrix. MFA also puts more weight on the GC matrix but less than SCA-P because the weights used by MFA (the largest singular value) correct both for size and redundancy: this reflects the larger heterogeneity in the GC matrix (LC measures mainly nucleotides while GC measures a larger variety of metabolites [4]). SUM-PCA gives equal weight to both matrices as it weights all specific matrices to unit sum of squares. Finally, the PCovR case where the cross-validation approach relies on reproducing the LC scores based on the observed GC scores, puts all weight on the LC data.

**Table 3:** Weights put on GC versus LC by different SCA methods.

	GC	LC
PCovR GC^1^	1.00	0
STATIS	.99	.01
SCA-P	.77	.23
MFA	.66	.34
SUM-PCA	.50	.50
PCovR LC^1^	0	1.00

^1^: Matrix-specific weights obtained by leave one out crossvalidation using the observed scores in the LC data to reproduce the scores of the GC data (PCovR GC) or the other way around (PCovR LC).

These weights have been calculated as the matrix-specific sum of squares divided by the sum of squares of the concatenated data.

To explore how dissimilar the common structure can be for the different methods (i.e., matrices **T **resulting from the different methods), we calculated Tucker's coefficient of congruence *ϕ *for pairs of matrices **T**, according to the definition below. For *R *components, Tucker's coefficient of congruence between two matrices of component scores **X **and **Y **is calculated as follows [28]:

(13)

with tr denoting the trace. (Here, *R *= 5 is taken throughout, because this is the number of components retained in the next section on the interpretation of the MFA solution). *ϕ *can be interpreted as an uncentered correlation and takes values between minus one and one (perfect congruence). It is invariant under scaling but not under rotation of the matrices **X **and **Y**. To account for the fact that PCA is invariant under reflection and rotation, but *ϕ *not, we will calculate (13) after applying a Procrustean similarity transformation without the translation step [29]. Except for STATIS, all methods are applied to the matrices obtained after autoscaling per metabolite; therefore we included the separate component analyses of the thus preprocessed data as a reference. The modified RV coefficient [26] calculated between preprocessed GC and LC matrices equals .20 (with 0 ≤ *RV *≤ 1) which is a low value such that the different weighting strategies can be expected to yield different results. Table 4 gives an overview of Tucker's congruence between the common scores obtained with the different simultaneous component methods and with the separate component analyses of the GC matrix and of the LC matrix (autoscaled per metabolite). A low congruence can be observed between STATIS and the other methods due to the fact that it relies on a different way of pre-processing. The two PCovR methods, which put all weight on either GC or LC, are perfectly congruent with GC and LC respectively and by consequence also have the same congruence with the other methods. For the methods applied to the data that are autoscaled per metabolite, it holds that methods that put more weight on GC/LC have higher congruence with the separate analysis of GC/LC respectively. When interest is in finding a simultaneous solution that is congruent with both separate analyses, SCA-P, MFA or SUM-PCA seem the methods of choice (for *R *= 5).

**Table 4:** Tucker's coefficient of congruence between the component scores (*R *= 5).

	PCovR GC	SCA-P	MFA	SUM-PCA	PCovR LC	LC^2^	STATIS
GC^1^	1	0.91	0.86	0.81	0.55	0.55	0.13
PCovR GC^3^		0.91	0.86	0.81	0.55	0.55	0.13
SCA-P			0.99	0.96	0.73	0.73	0.12
MFA				0.99	0.79	0.79	0.12
SUM-PCA					0.84	0.84	0.11
PCovR LC^4^						1	0.08
LC							0.08

^1^: Ordinary principal component analysis of GC data only

^2^: Ordinary principal component analysis of LC data only

^3+4^: Matrix-specific weights obtained by leave one out crossvalidation using the observed scores in the LC data to reproduce the scores of the GC data (PCovR GC) or the other way around (PCovR LC).

### Interpretation of the multiple factor analysis solution

**Figure 2:**
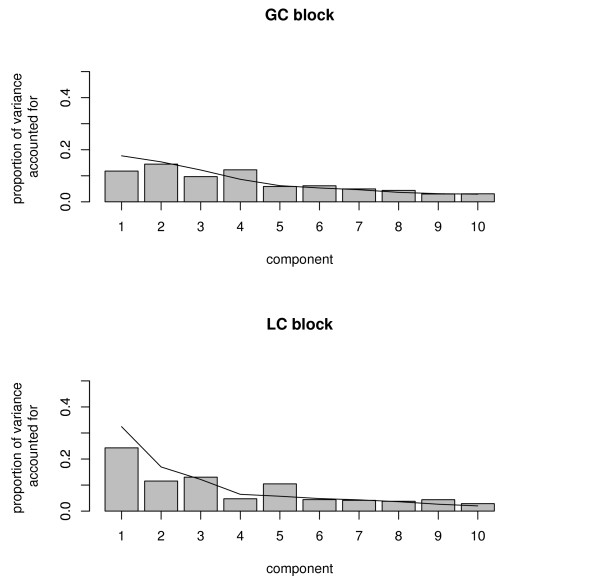
**Proportion of variance accounted for by the MFA solution**. Proportion of variance accounted for by the MFA solution in each matrix (bars) and proportion of variance accounted for by separate component analyses (lines).

In this section we will interpret the solution obtained with multiple factor analysis (MFA). Our motivation to choose this specific method is (1) that we want to attach equal importance to the biological processes behind the metabolites found by both types of separation methods so we need to correct for the difference in size between the matrices, and (2) that we do not want processes related to the many nucleotides in the LC data to dominate the solution. From Figure 2 it can be derived that five components are needed because the first five components account clearly for more variance than the next five in at least one of the data matrices.

For the interpretation of the components, we will first take a look at the scores of the samples on the components. We made use of the rotational freedom to rotate the scores to a simple structure using the VARIMAX criterion (see [30]): This is a rotation that targets a structure with high scores on only one component and low (close to zero) scores on the other components. Table 5 displays the resulting scores and, on the last three lines, the variance they account for overall (the 'TOTAL' line) and in each of the data blocks (the 'GC' and 'LC' lines). A description of the experimental design can be found in [3]; here it is summarized by the first column that labels the experiments in relation to the reference condition ('+' means more than in the reference, '-' less, and 'oxygen ?' means that not the dissolved oxygen level but the steering speed of the fermenter was controlled) and by the second column that reports after how many hours the samples were taken from the bioreactor. Taking a look at the two last lines of Table 5, we see that the first two components are involved in both types of separation methods while the third and fifth component seem to be specific for LC and the fourth for GC. Furthermore, the information captured in the different simultaneous components (SC1 to SC5) showed a clear link with the environmental conditions under which these samples were generated. For instance, based on this analysis SC1 seems to comprise metabolic processes related to oxygen limitation and to the early stationary growth phase (after approximately 40 hours of fermentation) which might be the result of oxygen stress and SC4 captures metabolic processes related to succinate catabolism (Table 5). This analysis also suggests that the metabolic processes related to mid-logarithmic (after approximately 24 hours of fermentation) to early stationary when grown at a higher pH or at a lower phosphate concentration than under the reference condition are similar: the samples taken under these conditions have a high component score on both SC1 and SC3. Moreover, the LC specific components are dominated by the samples taken from the fermentation performed with the wild type strain and that were performed at an elevated pH compared to the reference fermentation condition.

**Table 5:** Component scores (labeled 'SC1' to 'SC5') after VARIMAX rotation of the MFA solution with five components and, on the last three lines, the variance accounted for by these components in the GC data (the 'GC' line), the LC data (the 'LC' line), and the concatenated data (the 'TOTAL' line).

	SC1	SC2	SC3	SC4	SC5
**Reference**					
**16 hrs**	0.07	**0.33**	-0.04	0.01	-0.12
**24 hrs**	-0.05	-0.02	-0.10	-0.09	-0.16
**32 hrs**	-0.01	**-0.30**	-0.05	-0.07	-0.06
**40 hrs**	0.10	**-0.27**	-0.01	-0.09	0.01
**48 hrs**	0.18	-0.17	0.17	-0.03	0.10
**pH +**					
**16 hrs**	0.18	-0.11	**-0.29**	-0.10	**-0.61**
**24 hrs**	0.03	-0.07	**0.36**	-0.10	-0.03
**40 hrs**	**0.35**	-0.17	**0.26**	0.03	-0.09
**48 hrs**	**0.22**	-0.10	0.05	-0.10	0.03
**oxygen +**					
**40 hrs**	**-0.24**	0.04	0.02	0.00	-0.06
**oxygen ?**					
**16 hrs**	0.04	**0.34**	0.01	-0.11	-0.06
**24 hrs**	**-0.26**	-0.02	-0.02	-0.08	-0.16
**40 hrs**	**-0.45**	-0.13	0.09	-0.05	0.08
**64 hrs**	**-0.37**	-0.10	0.11	-0.02	0.07
**phosphate +**					
**16 hrs**	0.01	**0.38**	-0.02	-0.04	0.03
**24 hrs**	-0.09	**0.43**	0.04	-0.10	0.14
**40 hrs**	**-0.33**	0.00	-0.04	-0.01	-0.01
**48 hrs**	-0.04	-0.12	0.01	0.01	-0.15
**phosphate -**					
**16 hrs**	-0.03	0.06	0.00	-0.19	-0.09
**24 hrs**	-0.03	-0.02	**0.41**	-0.12	0.07
**40 hrs**	**0.32**	0.09	**0.32**	-0.01	0.14
**succinate**					
**24 hrs**	0.07	0.10	-0.02	**0.55**	-0.13
**40 hrs**	-0.01	-0.02	-0.01	**0.57**	-0.03
**48 hrs**	-0.05	-0.05	-0.05	**0.46**	0.11
**Wild type**					
**16 hrs**	0.19	**0.27**	**-0.25**	-0.10	0.06
**24 hrs**	0.02	-0.11	**-0.38**	-0.12	0.04
**40 hrs**	0.08	**-0.20**	**-0.32**	-0.06	**0.33**
**48 hrs**	0.11	-0.06	**-0.26**	-0.03	**0.55**

**GC**	0.14	0.12	0.08	0.14	0.06
**LC**	0.10	0.14	0.20	0.07	0.13
**TOTAL**	0.13	0.13	0.12	0.12	0.09

In the part with component scores, the first column describes the experiments in relation to the reference condition and the number of hours the samples were in the bioreactor. Component scores ≥ .25 (in absolute value) are in boldface.

**Figure 3:**
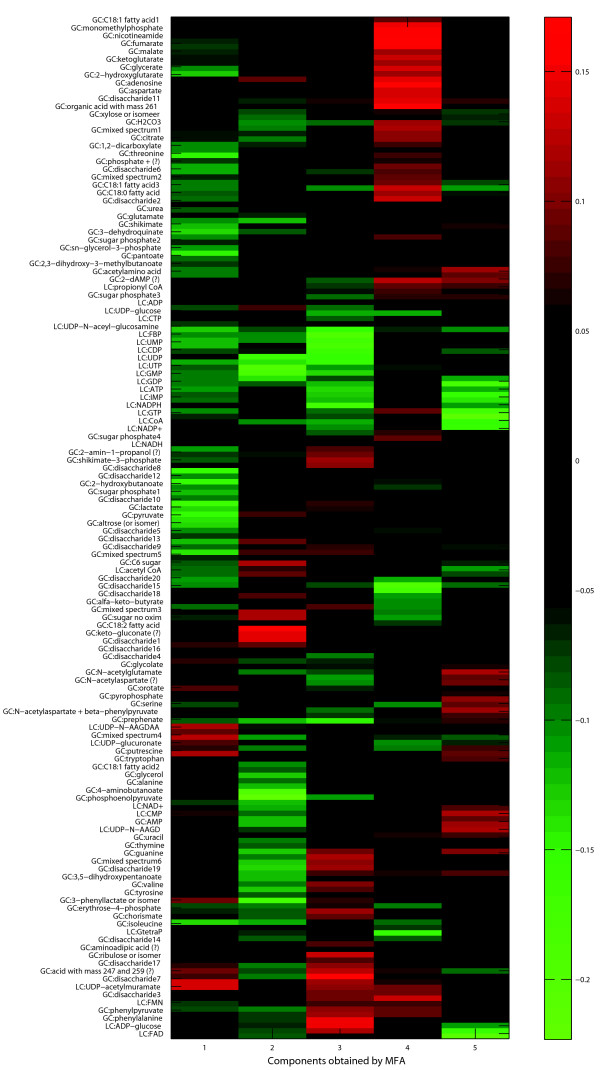
**Heatmap**. Heat map of the metabolite loadings on the five components. Labels were used for 130 of the 188 metabolites (the labels 'unknown' were dropped).

Figure 3 visualizes the relation between the 188 metabolites and the components through a so-called heatmap: the loading of the metabolites on each of the components is depicted by a color with bright red for strong positive scores, black for scores around zero, and bright green for very negative scores. The order of the metabolites was determined by a hierarchical clustering (average linkage) using one minus the cosine of the angle between the vectors of metabolite loadings as a distance measure. Note that we labeled 130 of the 188 metabolites. Different groups of metabolites could be identified whose concentration were higher or lower in samples obtained under specific environmental conditions. For instance, a first group of metabolites (i.e. fumarate, malate, aspartate, *α*-ketoglutarate, and 2-hydroxyglutarate) is characterized by positive loadings on SC4 (succinate catabolism): These metabolites are more abundant in samples with succinate as a carbon source. As fumarate and malate are the direct catabolic intermediates in the degradation of succinate this makes biological sense. Moreover, the increased 2-hydroxyglutarate concentrations suggest that part of the reducing equivalents formed during succinate catabolism are (partially) used to reduce *α*-ketoglutarate. A second example is composed out of a group of metabolites consisting out of N-acetylglutamate, N-acetylaspartate and *β*-phenylpyruvate. The concentrations of these metabolites are higher in the samples obtained from the wild type strain, and at the early logarithmic growth phase. By PLS analysis we have demonstrated that these compounds specifically correlated with the phenylalanine production titer (unpublished results), while the samples where the concentrations of these compounds are high reflect non-phenylalanine producing growth conditions and/or a non-phenylalanine overproducing strain.

## Discussion

In this paper we proposed a general framework for simultaneous component data integration methods based on six aspects: (1) the data, (2) pre-processing, (3) the model, (4) the objective function, (5) identification constraints, and (6) the algorithmic strategy. For two of these aspects there are no choices to be made that yield different results (namely the objective function and the algorithmic strategy). For the four remaining aspects, we discussed for each of the published simultaneous component methods (SUM-PCA, unrestricted PCovR, MFA, STATIS, and SCA-P) the specific choices they make. We further discussed which choices imply significant consequences for the obtained results:

1. Labeling the common mode as object versus variable is consequential for the obtained results only when considered in combination with pre-processing (the variable-wise autoscaling).

2. Pre-processing can have a strong influence on the obtained results. This is the case when the variables are measured on different scales or when the block-specific sum of squares differ substantially. In the former case a single/few variables can dominate the solution, in the latter a single/few matrices. Applying a simultaneous component analysis to data corrected for such differences may yield very different results from an analysis on the uncorrected data.

3. In general, different matrix-specific weighting strategies lead to different results. The different simultaneous component methods each proposed specific weighting strategies based on different principles of fairness. As illustrated, these strategies may yield very different weights ranging from one extreme (all weight is put on a particular matrix) to the other (all weight is put on another particular matrix).

4. The identification constraint to impose orthonormality on the common versus on the concatenated model structure affects the scale of the component scores and loadings but it is not consequential and yields the same reproduced scores.

## Conclusion

Summarizing, of the four aspects that differentiate the simultaneous component methods, the choices made with respect to pre-processing and weighting are consequential; this was also illustrated by applying the different simultaneous component methods to two coupled metabolomics data matrices. Especially, the type of weighting of the different matrices is essential for simultaneous component analysis. These types were shown to be linked to different specifications of the idea of a fair integration of the different coupled matrices. A summary overview of these principles (see Table 2) may be of help to the data analyst in choosing an appropriate weighting scheme for the analysis of a data set at hand. As discussed, such a weighting scheme may be based on a flexible integration of different principles of fairness.

## Abbreviations

GC: Gas Chromatography; GPA: Generalized Procrustes Analysis; LC: Liquid Chromatography; MFA: Multiple Factor Analysis; MS: Mass Spectrometry; PCovR: Principal Covariates Regression; PLS: Partial Least Squares; SCA-P: Simultaneous Component Analysis with a fixed Pattern matrix; STATIS: Structuration des Tableaux à Trois Indices de la Statistique; SVD: Singular Value Decomposition.

## Authors' contributions

KVD carried out the literature study, performed the statistical analyses and drafted the manuscript. AKS coordinated the statistical analysis of the metabolomics data. MJvdW provided these data and performed the biological interpretation. AKS, HALK, IVM, and KVD conceived of the study, and participated in its design and coordination. AKS, HALK, MJvdW, and especially IVM helped to draft the manuscript. All authors read and approved the final manuscript.

## Supplementary Material

Additional file 1 **Estimation of the simultaneous component scores and loadings**. Estimation.pdf describes how optimal parameters can be obtained either by a singular value decomposition of the concatenated weighted data matrices or by a two-step approach. It also contains a proof of their equivalence. Click here for file
