# Supplementary material for: A structured overview of simultaneous component based data integration
Source: BMC Bioinformatics. 2009 Aug 11;10:246. doi: 10.1186/1471-2105-10-246 (PMC2752463; doi:10.1186/1471-2105-10-246)
Supplement: Additional file 1 — Estimation of the simultaneous component scores and loadings. Estimation.pdf describes how optimal parameters can be obtained either by a singular value decomposition of the concatenated weighted data matrices or by a two-step approach. It also contains a proof of their equivalence. [file 1471-2105-10-246-S1.pdf]

Model estimation

In this document we show how optimal parameters that minimize the loss function associated to the simultaneous component methods can be obtained either by a singular value decomposition (SVD) of the concatenated weighted data matrices or by a two-step approach in which first the common structure is found by an eigendecomposition of the sum of cross-product matrices and second the K companion matrices are found by means of a suitable regression analysis.

The strategy that uses a singular value decomposition of the concatenated data, relies on the following equivalences: (1) In case of a common object (row) mode:

$$\min_{\mathbf{T},\mathbf{P}_k} \sum_k \|\mathbf{X}_k - \mathbf{T}\mathbf{P}_k^T\|^2 = \|\mathbf{X}_{concV} - \mathbf{T}\mathbf{P}_{conc}^T\|^2, \tag{1}$$

with $\mathbf{X}_{concV} = [w_1\mathbf{X}_1 \ldots w_k\mathbf{X}_k \ldots w_K\mathbf{X}_K]$ being of size $I \times (\sum_k J_k)$ and representing the matrix of the concatenated weighted data matrices and $\mathbf{P}_{conc} = [\mathbf{P}_1^T \ldots \mathbf{P}_K^T]^T$ of size $(\sum_k J_k) \times R$ representing the matrix of concatenated block specific loadings; (2) in case of a common variable (column) mode:

$$\min_{\mathbf{T}_k,\mathbf{P}} \|\mathbf{X}_{concO} - \mathbf{T}_{conc}\mathbf{P}^T\|^2, \tag{2}$$

with $\mathbf{X}_{concO} = [w_1\mathbf{X}_1^T \ldots w_K\mathbf{X}_K^T]^T$ being of size $(\sum_k I_k) \times J$ and $\mathbf{T}_{conc} = [\mathbf{T}_1^T \ldots \mathbf{T}_K^T]^T$, of size $(\sum_k I_k) \times R$, representing the matrix of concatenated block specific component scores.

In the first case a solution for the component scores and loadings can be obtained directly from an SVD of the concatenated data $\mathbf{X}_{concV}$, $\mathbf{X}_{concV} = \mathbf{U}\mathbf{S}\mathbf{V}^T = \mathbf{U}\mathbf{S}^p\mathbf{S}^{(1-p)}\mathbf{V}^T$. The R common component scores $\mathbf{T}$ can then be obtained by taking the R left singular vectors scaled by the associated R largest singular values raised to the power p,

$$\mathbf{T} = \mathbf{U}_R\mathbf{S}_R^p, \tag{3}$$

and the R loading vectors by taking the R right singular vectors scaled by the associated R largest singular values raised to the power $1-p$, $\mathbf{P}_{conc} = \mathbf{V}_R\mathbf{S}_R^{(1-p)}$. For the second case, a solution for the component scores and loadings can be obtained analogously, from an SVD of the now differently concatenated data $\mathbf{X}_{concO}$ in (6), $\mathbf{X}_{concO} = \mathbf{U}\mathbf{S}^p\mathbf{S}^{(1-p)}\mathbf{V}^T$, with the R common loadings given by

$$\mathbf{P} = \mathbf{V}_R\mathbf{S}_R^{(1-p)} \tag{4}$$

and the component scores by $\mathbf{T}_{conc} = \mathbf{U}_R\mathbf{S}_R^p$.

These component scores and loadings have a principal axes orientation; orthonormal component scores can be obtained by taking $p = 0$, while orthonormal loadings can be obtained by taking $p = 1$.

The second algorithmic strategy relies on a two-step approach where: (1) in the first step common component scores or common loadings are derived from an eigendecomposition of the matrix $\mathbf{X}_{concV}\mathbf{X}_{concV}^T = \sum_k w_k^2\mathbf{X}_k\mathbf{X}_k^T$ in the first case (resp. $\mathbf{X}_{concO}^T\mathbf{X}_{concO} = \sum_k w_k^2\mathbf{X}_k^T\mathbf{X}_k$ in the second case), and (2) in the second step concatenated loadings or component scores can be obtained by regressing $\mathbf{X}_{concV}$ on $\mathbf{T}$ (resp. $\mathbf{P}$): $\mathbf{P}_{conc} = \mathbf{X}_{concV}^T\mathbf{T}(\mathbf{T}^T\mathbf{T})^{-1}$ (resp. $\mathbf{T}_{conc} = \mathbf{X}_{concO}\mathbf{P}(\mathbf{P}^T\mathbf{P})^{-1}$). Note that these can also be obtained by concatenating the block specific regressions: $\mathbf{P}_k = \mathbf{X}_k^T\mathbf{T}(\mathbf{T}^T\mathbf{T})^{-1}$ (resp. $\mathbf{T}_k = \mathbf{X}_k\mathbf{P}(\mathbf{P}^T\mathbf{P})^{-1}$). In the first step, an orthonormal $\mathbf{T}$ (resp. $\mathbf{P}$) can be obtained by setting it equal to the first R eigenvectors while a columnwise orthonormal concatenated score (resp. loading) matrix will be obtained when these eigenvectors are scaled by the square root of the associated eigenvalues.

Both algoritmic strategies result in the same solution. This can be understood by noting that the left (resp. right) singular vectors of $\mathbf{X}_{concV}$ (resp. $\mathbf{X}_{concO}$) can be found by an eigendecomposition of $\mathbf{X}_{concV}\mathbf{X}_{concV}^T$ (resp. $\mathbf{X}_{concO}^T\mathbf{X}_{concO}$), thus resulting in the same common component scores $\mathbf{T}$ (resp. loadings $\mathbf{P}$); from the equality of $\mathbf{T}$ (resp. $\mathbf{P}$), the equality of $\mathbf{P}_{conc}$ (resp. $\mathbf{T}_{conc}$) follows.
